# Ethylene-responsive VviERF003 modulates glycosylated monoterpenoid synthesis by upregulating *VviGT14* in grapes

**DOI:** 10.1093/hr/uhae065

**Published:** 2024-02-28

**Authors:** Ya-Chen Wang, Yi Wei, Xiang-Yi Li, Hui-Min Zhang, Xiao Meng, Chang-Qing Duan, Qiu-Hong Pan

**Affiliations:** Center for Viticulture and Enology, College of Food Science and Nutritional Engineering, China Agricultural University, Beijing 100083, China; Key Laboratory of Viticulture and Enology, Ministry of Agriculture and Rural Affairs, Beijing 100083, China; Center for Viticulture and Enology, College of Food Science and Nutritional Engineering, China Agricultural University, Beijing 100083, China; Key Laboratory of Viticulture and Enology, Ministry of Agriculture and Rural Affairs, Beijing 100083, China; Center for Viticulture and Enology, College of Food Science and Nutritional Engineering, China Agricultural University, Beijing 100083, China; Department of Plant Science, School of Agriculture and Biology, Shanghai Jiao Tong University, Shanghai 200240, China; Center for Viticulture and Enology, College of Food Science and Nutritional Engineering, China Agricultural University, Beijing 100083, China; Key Laboratory of Viticulture and Enology, Ministry of Agriculture and Rural Affairs, Beijing 100083, China; Center for Viticulture and Enology, College of Food Science and Nutritional Engineering, China Agricultural University, Beijing 100083, China; Key Laboratory of Viticulture and Enology, Ministry of Agriculture and Rural Affairs, Beijing 100083, China; Center for Viticulture and Enology, College of Food Science and Nutritional Engineering, China Agricultural University, Beijing 100083, China; Key Laboratory of Viticulture and Enology, Ministry of Agriculture and Rural Affairs, Beijing 100083, China; Center for Viticulture and Enology, College of Food Science and Nutritional Engineering, China Agricultural University, Beijing 100083, China; Key Laboratory of Viticulture and Enology, Ministry of Agriculture and Rural Affairs, Beijing 100083, China

## Abstract

Terpenoids are important contributors to the aroma of grapes and wines. Grapes contain terpenoids in both volatile free form and non-volatile glycosidic form, with the latter being more abundant. Glycosylated terpenoids are deemed as latent aromatic potentials for their essential role in adding to the flowery and fruity bouquet of wines. However, the transcriptional regulatory mechanism underlying glycosylated terpenoid biosynthesis remains poorly understood. Our prior study identified an AP2/ERF transcription factor, VviERF003, through DNA pull-down screening using the promoter of terpenoid glycosyltransferase VviGT14 gene. This study demonstrated that both genes were co-expressed and synchronized with the accumulation of glycosylated monoterpenoids during grape maturation. VviERF003 can bind to the *VviGT14* promoter and promote its activity according to yeast one-hybrid and dual-luciferase assays. VviERF003 upregulated *VviGT14* expression in vivo, leading to increased production of glycosylated monoterpenoids based on the evidence from overexpression or RNA interference in leaves, berry skins, and calli of grapes, as well as tomato fruits. Additionally, *VviERF003* and *VviGT14* expressions and glycosylated monoterpenoid levels were induced by ethylene in grapes. The findings suggest that VviERF003 is ethylene-responsive and stimulates glycosylated monoterpenoid biosynthesis through upregulating *VviGT14* expression.

## Introduction

Terpenoids, comprising more than 40 000 components, are the most abundant group of secondary metabolites. They are subdivided into monoterpenoids, sesquiterpenoids, diterpenoids, and triterpenoids based on the count of carbon atoms [1]. Terpenoids have important biological functions in plant interaction with the environment, including signaling between plants, pollinators, and herbivores, as well as defense against biotic and abiotic stresses [2, 3]. Additionally, terpenoids, especially monoterpenoids and sesquiterpenoids, are significant contributors to the floral and fruity aroma of fruits and their processed products. In grapes, monoterpenoids exceed 80% of the total terpenoid content, with linalool and geraniol being the most significant ones that lend the attributes of rose, floral, and citrus fragrance to wine [1].

Both free and glycosidically bound forms of terpenoids occur in grapes, with the latter being more abundant. The concentration of glycosylated monoterpenoids in grapes is approximately 2–8 times higher than that of the free form [4, 5]. Glycosylation of terpenoids alters their solubility, biological activity, and membrane translocation [6]. Unlike their free counterparts, which are volatility, glycosylated terpenoids are more probable to be transferred into the vacuoles of grape berry cells, where they are stably stored in a water-soluble state [3, 7]. Furthermore, glycosylated terpenoids present in grapes possess the potential to serve as significant aroma components in wine due to their ability to release free-form terpenoids gradually through chemical or enzymatic hydrolysis during the vinification process [5, 8].

In plant, prenyltransferases catalyze IPP and DMAPP to generate prenyl diphosphate, which is a central precursor of various types of terpenoids. After synthesizing different C_5n_ prenyl diphosphates, terpenoid synthases (TPSs) catalyze the generation of linear or cyclic terpene olefins and terpene alcohols with diverse structures [2]. Additionally, cytochrome P450 oxygenases (CYPs), dioxygenases or dehydrogenases can further oxidize the production at specific positions and in a stereospecific manner [9]. The primary terpenoids can then be modified by the addition of various substituents using enzymes like hydroxylases, dehydrogenases, reductases, methyltransferases, and glycosyltransferases [10]. Glycosyltransferases (GTs) catalyze the transfer of sugar moiety to the aglycone acceptor, forming glycosides. Proteins belonging to the GT family 1 typically utilize uridine diphosphate-alpha-D-Glucose (UDPG) as the glycogen donor and are referred to as uridine diphosphate glycosyltransferases (UGTs) [11, 12].

UGTs are associated with the glycosylation of numerous important secondary metabolites, including flavonoids [13], anthocyanins [14], and terpenoids [15]. Several UGTs have been biochemically characterized in grapes for the formation of glycosylated terpenoids. Bonisch *et al.* confirmed that VviGT14, VvGT15a, VvGT15b, and VvGT15c can glucosylate geraniol, *R*, *S*-citronellol, and nerol [12]. The expression of *VviGT7* is linked to the accumulation of geranyl and neryl glucosides during grape maturation [16]. The mutations in the *VviGT7* alleles may cause the changes in the enzyme activity of monoterpenoid glycosylation in different grape varieties [15]. Our previous study has demonstrated a significant positive correlation between the expression of *VviGT14* and the differential accumulation of monoterpenyl glycosides in *Vitis vinifera* L. Muscat blanc à Petit grain grapes from two distinct wine regions in China with different climates [4]. Compared to the research progress on glycosylated terpenoids and UGTs in grapes, the transcriptional regulatory mechanism of glycosylated terpenoid biosynthesis is of lesser concern.

Terpenoid biosynthesis is regulated by various factors, such as environmental conditions, phytohormones, and transcription factors [17]. Transcriptional regulation significantly impacts the biosynthesis of terpenoids. Several transcription factor families, including WRKY, MYB, bHLH, bZIP, and AP2/ERF, play an important role in terpenoid synthesis in plants. In *Arabidopsis thaliana*, AtMYC2 binds to the promoters of *AtTPS21* and *AtTPS11,* triggering their expression and increasing sesquiterpenoid emission levels [18]. In flowers of *Freesia hybrida*, Yang *et al.* revealed the function of MYB21 and MYC2 in linalool biosynthesis [19]. In addition, Chuang *et al*. found that *PbbZIP4* overexpression resulted in increased production of monoterpenoids in scentless orchids [20]. Several plant hormones, such as jasmonates, abscisic acid (ABA), and ethylene, regulate terpenoid accumulation [17, 21, 22]. The application of exogenous ethylene and the increase in endogenous ethylene production can promote terpenoid accumulation in fruits [21, 23]. Ethylene response factors of the AP2/ERF family have been identified to play a role in the regulation of terpenoid biosynthesis. *Zea mays* EREB58 [24], *Artemisia annua* AaERF1 and AaERF2 [25], and *Citrus* CitERF71 [26] have been proven to participate in the transcriptional regulation of terpenoid synthesis. While the impact of transcriptional regulation in terpenoid synthesis is well established, less attention has been given to its involvement in terpenoid glycosidation.

**Figure 1:**
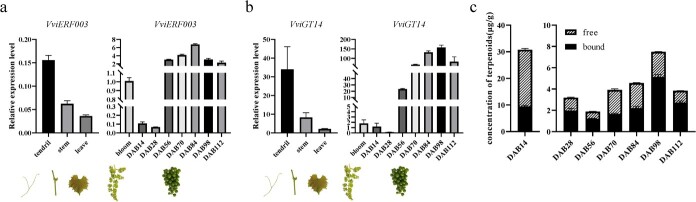
Spatiotemporal expression patterns of *VviERF003***a** and *VviGT14***b** and change in total concentrations of free and glycosylated monoterpenoids **c** in different tissues of ‘Muscat blanc à Petit grain’ grapevines.

Our prior research deployed the *VviGT14* promoter as a bait for conducting DNA pull-down analysis, and identified 10 expected transcription factors, among which VviWRKY40 was found to negatively regulate *VviGT14* expression, thereby impeding the accumulation of glycosylated monoterpenoids in grapes [27]. However, the mechanism behind positive transcriptional regulation of glycosylated terpenoid synthesis in grapes remains uncertain. Research has shown that AP2/ERF transcription factors have a positive regulatory function in terpenoid synthesis [28, 29]. This study investigated a specific member of the AP2/ERF family, VviERF003, which was found to bind with the promoter of *VviGT14* under DNA pull-down conditions. The function of VviERF003 in regulating glycosylated monoterpenoid production was proposed. The findings supplement the transcriptional regulatory network of monoterpenoid glycosidation in grapes.

## Result

### *VviERF003* and *VviGT14* exhibit co-expression patterns

Our prior research indicated that VviERF003 binds to the promoter of *VviGT14* under DNA pull-down [27]. This study investigated the spatiotemporal expression of *VviERF003* and *VviGT14* and found co-expression patterns in grapevine, notably in maturing grape berries. During fruit development, the expressions of both *VviERF003* and *VviGT14* decreased in the early stages and then increased dramatically at 56 days after bloom (DAB) (véraison). They were subsequently maintained at high levels during the period of berry ripening (Fig. 1a and b). In comparison, *VviERF003* exhibited low expression levels in tendrils, stems, and leaves. Using Pearson’s correlation analysis, a significantly positive correlation was identified between *VviGT14* and *VviERF003* expressions (Pearson’s coefficient = 0.772, *P* = 0.0053), as shown in the Fig. S1 (see online supplementary material).

**Figure 2:**
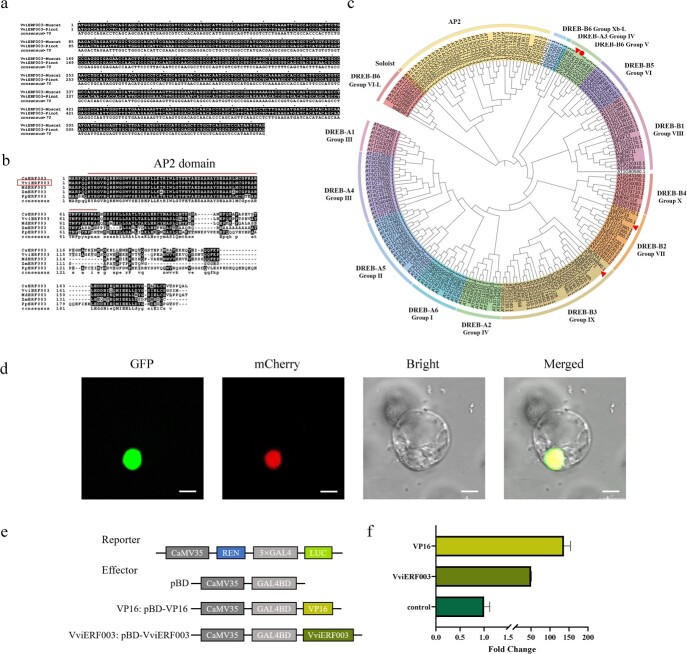
Sequence analysis, nuclear localization, and transcription activation of VviERF003. **a** Gene sequence alignment of *VviERF003* in grapes of different aromatic varieties. **b** Multiple amino acid sequence alignment of VviERF003 and homologous proteins from citrus, apple, maize, and peach. The alignments were performed using ClustalX. Black and light gray shading indicate identical and conserved amino acid residues, respectively. The line shows the AP2/ERF domain. **c** Phylogenetic analysis of VviERF003 and other AP2/ERF family proteins from other species with known functions or *Arabidopsis*. The phylogenic tree was constructed based on amino acid sequence using the neighbor-joining method with 1000 bootstrap replicates by MEGA 6. The circle indicates VviERF003. The triangles indicate VviERF045, CitERF71, and PjERF1. **d** Subcellular localization of VviERF003 in *Arabidopsis* protoplasts. AtFBI-mCherry was used as a nuclear marker. Bar = 10 μm. **e** Schematic diagrams of vectors used for transcriptional activity assay. **f** Transactivation property assay of VviERF003 in *Arabidopsis* protoplasts. The control was set as 1 to be a calibrator. VP16 was used as a positive control.

A total of 32 monoterpenoids were identified and quantified and they included both free and glycosylated forms. Most of these compounds showed an overall increase in concentrations from véraison to maturity or harvest, with DAB98 exhibiting the highest concentrations listed in Tables S1 and S2 (see online supplementary material). The concentration of glycosylated monoterpenoids was significantly greater than that of free-form monoterpenoids at maturity (DAB98) and harvest (DAB112) (Fig. 1c). During berry development, glycosylated monoterpenoids showed high levels at the beginning of fruit development and subsequently decreased gradually until véraison. Following that, their concentrations increased along with berry maturation. As a result, the total monoterpenoids, which comprise glycosylated and free forms, followed a trend that was more aligned with the change in glycosylated monoterpenoids (Fig. 1c; Table S1, see online supplementary material). Similar trends were observed in the concentrations of monoterpenoids and the expression levels of *VviERF003* and *VviGT14*, as shown in Fig. 1 and Tables S1 and S2 (see online supplementary material). These results suggest that VviERF003 may positively regulate the expression of *VviGT14* and accumulation of glycosylated monoterpenoids during grape development.

### VviERF003 is nuclear-localized and possesses transcription activation activity

The CDS of VviERF003 was isolated from berries of ‘Muscat blanc à Petit grain’, and is 564 bp in length, encoding 188 amino acids. Aligning gene sequence showed that the sequence of *VviERF003* is identical in both the Muscat-type variety ‘Muscat blanc à Petit grain’ and non-aromatic variety ‘Pinot Noir’ (Fig. 2a). A multiple sequence alignment illustrated that VviERF003 was similar to other AP2/ERFs and featured a single AP2 domain (at positions 7 to 69) (Fig. 2b), which is characteristic of the AP2/ERF family of transcription factors [30]. According to a phylogenetic tree, VviERF003 is a part of the group V subfamily and in the same clade as VviERF045, which has been linked to grape maturation [31]. However, it was determined that VviERF003 was distantly related to other AP2/ERF transcription factors that have been reported to regulate terpenoid biosynthesis, such as CitERF71 and PjERF1 (Fig. 2c), belonging to the group IX, VII subfamily, respectively [26, 29].

**Figure 3:**
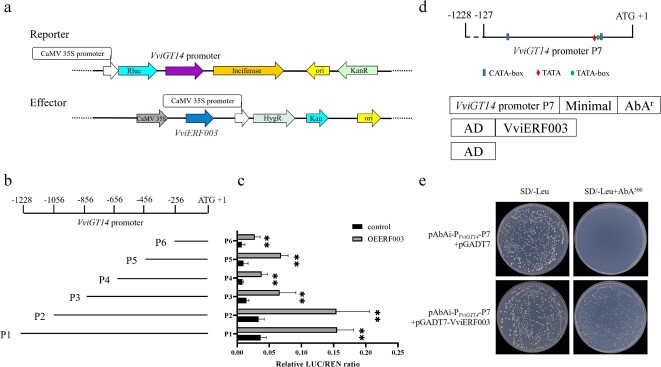
VviERF003 binds to the promoter of *VviGT14* and activates the expression of *VviGT14*. **a** Schematic diagram of the effector and reporter constructs used for the dual-luciferase assay. **b** Schematic diagram of *VviGT14* promoter segmentation. **c** Dual-luciferase assay showing relative VviERF003 activation of different promoter fragments of *VviGT14*. Asterisk indicates statistical significance using independent sample *t*-test, ^**^P < 0.01. **d** Schematic diagram of the constructs used for the yeast one-hybrid assay. **e** The binding analysis of VviERF003 to the promoter fragments P7 of *VviGT14* by yeast one-hybrid assay*.*

To ascertain the functionality of VviERF003 as a transcription factor, GFP was fused with VviERF003 and transiently co-expressed with the nuclear maker AtFBI-mCherry in *Arabidopsis* protoplasts. Fluorescence observations revealed the GFP signals overlapped with the mCherry signals, indicating colocalization of the VviERF003-GFP fusion protein with AtFBI-mCherry in the nucleus. This indicates that VviERF003 can perform its function in the nucleus (Fig. 2d).

To determine whether VviERF003 possesses transcriptional activation ability, the GAL4 DNA-binding domain was merged with the *VviERF003* gene vector as an effector, while the VP16 transcriptional activation domain was employed as a positive control. These constructs were co-expressed with a reporter vector featuring 3 × GAL4 activation domains upstream of the *LUC* gene and the *REN* gene, both governed by the CaMV35S promoter, in *Arabidopsis* protoplasts. The results demonstrated that both pBD-VviERF003 and pBD-VP16 significantly elevated relative luciferase activity as compared to the negative control pBD, indicating the transcriptional activation activity of VviERF003 *in vivo* (Fig. 2e and f).

### VviERF003 binds directly to *VviGT14* promoter and stimulates its activity

To determine if *VviGT14* is a target gene of VviERF003, we constructed the report vectors with truncated fragments of the *VviGT14* promoter, the goal of which is to confirm the action region of VviERF003 on the *VviGT14* promoter (Fig. 3a). The dual-luciferase assay revealed that VviERF003 had trans-activation effect on the promoter of *VviGT14*. Furthermore, when the *VviGT14* promoter was truncated to P2–P6, the trans-activation effect was still observed, providing evidence that VviERF003 activates the transcription of *VviGT14*. This suggests the existence of an action site for VviERF003 on P6 (Fig. 3b and c).

Because VviERF003 was identified via DNA pull-down screening using the *VviGT14* promoter as the bait, it can be inferred that VviERF003 binds to the promoter of *VviGT14*. To further confirm the existence of a direct binding effect, we conducted a Y1H assay. Because the AP2/ERF family binding motif or its variants were not present on the *VviGT14* promoter, we truncated the promoter to 127 bp upstream of the start codon (P7) (Fig. 3d; Fig. S2, see online supplementary material). Bait yeast cells with the promoter fragments P7, along with the control vector (AD) or the fusion vector (AD-VviERF003) showed steady growth on synthetic dropout medium (SD) without Leu. However, only the yeast cells that were co-transformed with the fusion vector AD-VviERF003 survived on the selected medium supplemented with Aureobasidin A (AbA) at a working concentration of 500 ng/mL (Fig. 3e). These results suggest that the VviERF003 protein can directly bind to the promoter fragment P7 of *VviGT14*, although the exact motifs involved are unknown.

### *VviERF003* overexpression promotes *VviGT14* expression and glycosylated monoterpenoid accumulation

**Figure 4:**
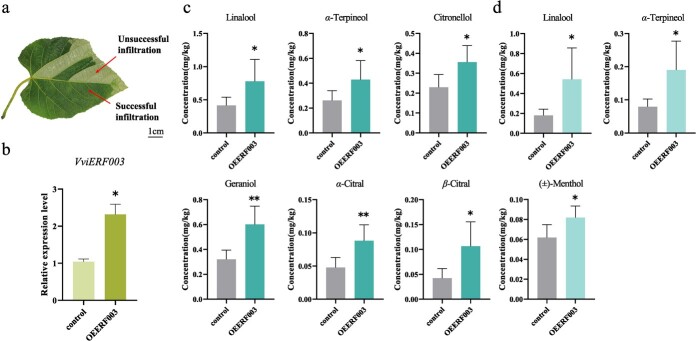
Effects of transient expression of *VviERF003* in ‘Yeniang-2’ leaves. **a** Phenotypes of transgenic leaves. Only successful infiltration part was used for subsequent analysis. **b** Relative expression levels of *VviERF003*, **c** concentrations of glycosylated and **d** free-form monoterpenoids relative in transgenic leaves. Asterisk indicates statistical significance using independent sample *t*-test, ^*^P < 0.05, ^**^P < 0.01.

To investigate the regulation of glycosylated monoterpenoid production by VviERF003, we transiently overexpressed it in grape leaves of *Vitis quinquangularis* Yeniang-2 variety and harvested the dark sections of the transformed leaves (Fig. 4a and b). The expression level of *VqGT7* was significantly increased in *VviERF003*-overexpression grape leaves, while the expression levels of *VqGT14* and *VqGT15* were not affected (Fig. S3a, see onlinesupplementary material). We quantified a total of 18 glycosylated monoterpenoids and 10 free-form monoterpenoids in the ‘Yeniang-2’ leaves using GC–MS (Fig. 4c and d; Fig. S3b and c, see online supplementary material). Glycosylated forms of linalool, *α*-terpineol, citronellol, *β*-citral, and geraniol exhibited a significant increase in the leaves overexpressing *VviERF003* (Fig. 4c), while the free-forms of linalool, *α*-terpineol, and (±)-menthol were also enhanced (Fig. 4d). These results suggest that VviERF003 may positively regulate glycosylated monoterpenoid biosynthesis.

**Figure 5:**
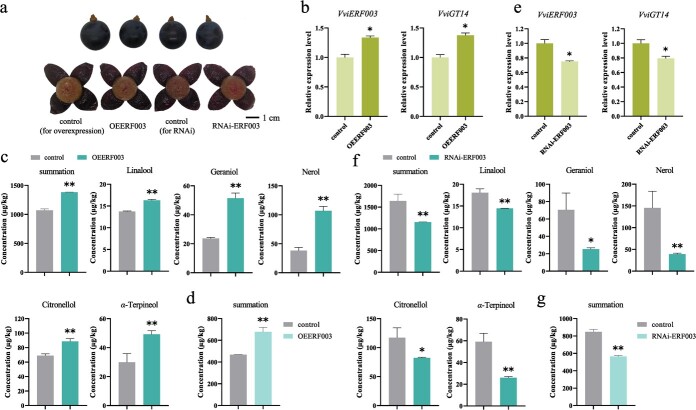
Effects of transient expression of *VviERF003* in ‘Summer Black’ berry skins. **a** Pictures of grapes taken from different treatment groups in transient experiment. The whole berries at the top were taken from different treatment groups before treatment, and the peeled berries taken from different treatment groups after treatment were at the bottom. Grape skins were collected for subsequent analysis. **b** Effects of *VviERF003* overexpression on the expression levels of genes, and **c** the concentrations of glycosylated and **d** free-form monoterpenoids in grape skins. **e** Effects of VviERF003 interfered expression on the expression levels of genes and **f** the concentrations of glycosylated and **g** free-form monoterpenoids in grape skins. Asterisk indicates statistical significance using independent sample *t*-test, ^*^P < 0.05; ^**^P < 0.01.

Furthermore, we conducted transient overexpression and silencing experiments of *VviERF003* in *V. vinifera* L. × *Vitis. labrusca* L. Summer Black grapes (Fig. 5a). The overexpression of *VviERF003* resulted in a significant increase in the expression level of *VviGT14* in grape skins (Fig. 5b). Subsequently, we analysed the concentrations of monoterpenoids in grape skins and quantified 29 glycosylated and 26 free-form monoterpenoids. With the exception of (±)-menthol, the concentrations of most glycosylated monoterpenoids, such as linalool, geraniol, nerol, citronellol, and the others, were significantly higher in grape skins overexpressing *VviERF003* compared to control grape skins (Fig. 5c; Fig. S4a, see online supplementary material). Additionally, *VviERF003* overexpression significantly increased the levels of most free-form monoterpenoids (Fig. 5d; Table S3, see online supplementary material). On the other hand, the interference of *VviERF003* expression led to a reduced expression level of *VviGT14* (Fig. 5e). The grape skins with suppressed *VviERF003* expression had lower levels of all detected glycosylated monoterpenoids, except for glycosylated (±)-menthol, compared to the control (Fig. 5f; Fig. S4b, see online supplementary material). Meanwhile, most of the free-form monoterpenoids were decreased, particularly citronellol, nerol, geraniol, *α*-phellandrene, cis-*β*-ocimene, and rose oxide, which were the most abundant monoterpenoids (Fig. 5g; Table S3, see online supplementary material).

**Figure 6:**
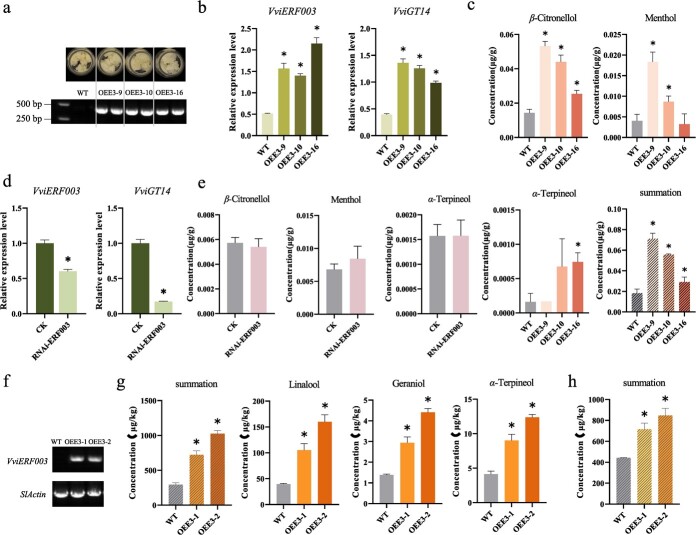
Effects of *VviERF003* in transgenic ‘Cabernet Sauvignon’ grape calli and tomato. **a** Phenotypes of transgenic calli and detection of hygromycin gene by PCR. **b** Effects of *VviERF003* overexpression in grape calli on relative expression of genes and **c** concentrations of glycosylated monoterpenoid compounds. **d** Changes in relative expression of *VviERF003* and *VviGT14,***e** concentrations of total monoterpenoids after enzymatic hydrolysis in calli in which *VviERF003* expression is transiently interfered. **f** Detection of *VviERF003*, *SlActin* genes by PCR and **g** concentrations of glycosylated and **h **free-form monoterpenoid compounds in WT and transgenic tomato fruits. Asterisk indicates statistical significance using independent sample *t*-test, ^*^P < 0.05.

We also investigated the *VviGT14* expression and monoterpenoid concentrations in the *V. vinifera* L. Cabernet Sauvignon calli with stably overexpression of *VviERF003*. Three independent transgenic lines (OEE3–9, OEE3–10, OEE3–16) with significant upregulated expression of *VviGT14* were assessed (Fig. 6a and b). Only three monoterpenoid compounds—*β*-citronellol, *α*-terpineol, and menthol—were identified in the calli with *β*-citronellol having the highest concentration, followed by menthol. The concentrations of total free-form and glycosylated *β*-citronellol and menthol were significantly higher in the *VviERF003*-overexpression calli than in the wild calli, while *α*-terpineol levels remained unaffected (Fig. S5a, see online supplementary material). Both *β*-citronellol and menthol, in either their free or glycosylated forms, exhibited increased concentrations in the transgenic lines. The overexpression of *VviERF003* significantly elevated the concentrations of glycosylated *β*-citronellol, menthol, and the sum of glycosylated monoterpenoids in the transgenic calli (Fig. 6c). The total concentration of three monoterpenoids in free-form significantly increased in OEE3–9 and OEE3–10 lines, but displayed no significant difference or lowered concentration in OEE3–16 (Fig. S5b, see online supplementary material). On the other hand, interference with *VviERF003* expression in grape calli resulted in a significant reduction of *VviGT14* expression (Fig. 6d). However, it was observed that there was no alteration in the concentrations of both free form and total monoterpenoid compounds, perhaps due to the low concentrations of terpenoids present in grape calli themselves (Fig. 6e; Fig. S5c, see online supplementary material).

Similar effects were observed in two transgenic tomatoes lines OEE3–1 and OEE3–2 with stable overexpression of *VviERF003* (Fig. 6f). This study quantified a total of 26 glycosylated and 17 free-form monoterpenoids, with most components, such as linalool, geraniol, and *α*-terpineol, showing significant increases in the transgenic fruits compared to wild-type tomato fruits (Fig. 6g and h; Table S4, see online supplementary material).

### Ethylene induces *VviERF003* and *VviGT14* expressions and glycosylated monoterpenoid production

**Figure 7:**
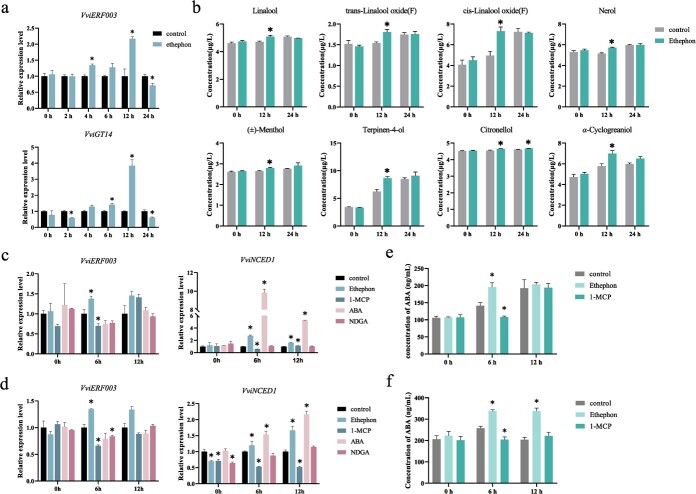
Expression of related genes and monoterpenoid contents after hormone treatment in grape berries. **a** The relative expression of related genes and **b** the concentrations of glycosylated monoterpenoids in ‘Muscat blanc à Petit grain’ berries before véraison under ethephon treatment. **c** Effects of ethephon, 1-MCP, ABA, and NDGA treatments, respectively, on the relative expression of related genes in ‘Muscat blanc à Petit grain’ berries at véraison, and **d** in ‘Jingxiangyu’ berries at véraison. **e** Effects of ethephon and 1-MCP on the concentrations of ABA in ‘Muscat blanc à Petit grain’ berries at véraison, and **f** in ‘Jingxiangyu’ berries at véraison. Asterisk indicates statistical significance using independent sample *t*-test, ^*^P < 0.05.

To determine if the expression of *VviERF003* is triggered by ethylene, we administered ethephon to ‘Muscat blanc à Petit grain’ grape berries about a week before véraison, and measured gene expression and monoterpenoid concentrations. Both *VviERF003* and *VviGT14* showed significantly increased expressions at 12 h post-treatment (Fig. 7a). Correspondingly, both *VviACO1*, a crucial gene associated with ethylene production, and the ethylene receptor gene *VviETR2* were upregulated (Fig. S6a, see online supplementary material). Further, 13 monoterpenoid compounds were detected in the processed berries, with eight glycosylated monoterpenoid compounds such as linalool, citronellol, and nerol significantly increasing at 12 h following ethephon treatment, as shown in Fig. 7b. The concentrations of other glycosylated monoterpenoids including *α*-terpineol, geraniol showed no significant changes (Fig. S6b, see online supplementary material). Meanwhile, free-form monoterpenoids displayed either an increasing or decreasing effect. Ethephon treatment significantly increased the concentrations of six free-form monoterpenoids such as linalool and *α*-terpineol, while decreasing the concentrations of five free-form monoterpenoids including citronellol, nerol, and geraniol (Fig. S6c, see online supplementary material).

To further confirm this observation, we subjected the *V. vinifera* L. Jingxiangyu and ‘Muscat blanc à Petit grain’ grapes to *in vitro* treatment with ethephon and the inhibitor of ethylene reception 1-methylcyclopropene (1-MCP) at véraison. The results indicated that the treatments with ethephon and 1-MCP increased and decreased the expression of the endogenous ethylene synthesis related gene *VviACO1* at 6 h post-treatment, respectively (Fig. S7, see online supplementary material). The expressions of *VviERF003* and the ethylene receptor gene *VviETR2* were significantly upregulated in the grapes treated with ethephon at 6 h post-treatment, and the promoting effect on *VviETR2* continued until 12 h after treatment. In contrast, both genes were downregulated in the 1-MCP-treated grapes at 6 h post-treatment (Fig. 7c and d; Fig. S7, see online supplementary material). These findings suggest that ethylene induces the expression of *VviERF003* and *VviGT14*, thereby enhancing the production of glycosylated monoterpenoids.

### ABA has no effect on *VviERF003* expression

The function of ABA in regulating terpenoid accumulation has been proven [22, 27]. Our prior investigation observed that ABA suppresses *VviWRKY40* expression, but stimulates *VviGT14* expression and glycosylated terpenoid production [27]. To investigate whether ABA plays a part in the regulation of VviERF003 on *VviGT14*, we conducted *in vitro* treatments of ABA and the ABA biosynthesis inhibitor snordihydroguaiaretic acid (NDGA) during véraison on ‘Jingxiangyu’ and ‘Muscat blanc à Petit grain’ grapes. The results demonstrated that ABA treatment increased the expression of *VviNCED1* and had no effect on the expression of *VviERF003,* indicating no responsiveness of *VviERF003* to ABA induction. The administration of NDGA did not affect *VviNCED1* and *VviERF003* expression (Fig. 7c and d). In contrast, ABA and NDGA treatments had varying effects on *VviETR2* in both varieties (Fig. S7, see online supplementary material). Ethephon treatment significantly elevated the level of *VviNCED1* expression at 6–12 h after treatment and increased the concentration of endogenous ABA at 6 h, while 1-MCP treatment had the opposite effect (Fig. 7c–f). At 12 h after 1-MCP treatment, the expression of *VviNCED1* significantly increased in ‘Muscat blanc à Petit grain’. However, the expression levels of *VviACO1* and *VviETR2* remained unaffected, indicating that endogenous ethylene was not impacted at this time (Fig. 7c; Fig. S7a, see online supplementary material). Based on these results, we propose that ABA does not have a regulatory influence on *VviERF003,* despite the fact that ethylene induces endogenous ABA synthesis.

## Discussion

Terpenoids are essential secondary metabolites in plants, serving as a means of plant resistance to biotic or abiotic stresses. Additionally, they possess high commercial value. Studies indicate that the majority of terpenoids in grapes bind to sugars and can be released during the winemaking and the aging process, intensifying the aromas of wine [16, 32]. Glycosylation boosts the stability and water solubility of terpenoids, thereby facilitating their preservation. Glycosylated terpenoids are highly regarded as potential contributors to wine aromas [5]. Our previous study revealed a significant correlation between the expression of *VviGT14* and the accumulation of glycosylated monoterpenoids in the Muscat-type grapevine varieties [4]. This study identifies VviERF003, an AP2/ERF transcription factor that is ethylene-induced and works as a positive regulator of glycosylated monoterpenoid biosynthesis by targeting *VviGT14* in grapes.

The AP2/ERF family of transcription factors play a crucial role in various aspects of plant physiology, such as growth, development, responses to stress, and the production of secondary metabolites such as flavonoids, anthocyanins, and alkaloids [33–39]. In grape, it has been proven that VvERF63 positively regulates the cold tolerance of leaves [40]. VvERF17 and VvERF75 regulate chlorophyll degradation by activating chlorophyll catabolic genes [41, 42]. Additionally, research has shown the involvement of AP2/ERF transcription factors in terpenoid biosynthesis. CitAP2.10 targets *CsTPS1* and upregulates the biosynthesis of (+)-valencene in oranges [21]. In *Panax japonicas*, PjERF1 has been found to promote triterpenoid biosynthesis though controlling the expression levels of key enzyme genes related to the biosynthesis of triterpenoid saponins [29]. It is worth noting that the previous studies have largely focused on free or total terpenoids, while our present study specifically investigates the regulation of AP2/ERF transcription factors on glycosylated monoterpenoid biosynthesis. The evidence indicates that VviERF003 functions as a transcriptional activator for the production of glycosylated monoterpenoids (Figs 1 and 4–6).

In plants, several studies have shown that AP2/ERF transcription factors participate in regulating the expression of genes related to terpenoid biosynthesis, including those involved in the MEP pathway, *TPSs*, *AaADS* [21, 25, 26], and *MdAFS* [43] were positively regulated by AP2/ERF transcription factors. Moreover, Wang *et al.* discovered that *Litsea cubeba* LcERF19 stimulates the production of geranial and neral by enhancing *LcTPS42* expression [28]. Terpenoids can be modified by oxidation, glycosylation, or hydroxylation following synthesis, thereby producing a wider array of components [3, 9]. Certain studies suggest that genes encoding post-modifying enzyme act as targets for AP2/ERF transcription factors. In *A. annua*, AaERF1 and AaERF2 were confirmed to enhance the expression of both *CYP71AV1* and *ADS*, resulting in an increased artemisinin concentration [25]. In grapevine, it has been established that *VviGT14* takes part in the glycosylation of monoterpenoids [12]. The study shows that *VviGT14* shares a similar spatiotemporal expression pattern with *VviERF003*, with both genes being highly expressed during grape maturation (Fig. 1a and b). Both *in vitro* and *in vivo* evidences indicate that VviERF003 has a positive impact on the production of glycosylated monoterpenoids by specifically targeting *VviGT14* (Figs 3, 5, and 6). The binding domain of AP/ERF transcription factors with the promoter of the target gene has been identified as either the GCC-box or DRE/CRT element. In particular, *Vitis amurensis* ‘Shuang You’ VaERF16 enhances the *VaPDF1.2* promoter activity by directly binding to the GCC-box [44]. Peach PpERF61 binds to the promoters of *PpTPS1* and *PpTPS3* through the DRE/CRT element [23]. No GCC-box or DRE element is present in the *VviGT14* promoter (Fig. S2, see online supplementary material). Recent research suggests that AP2/ERF transcription factors may attach to other elements beyond GCC-box and DRE, such as GCC-like motifs [45] or (A/G)CC(G/C)AC and AA(T)TTCAAA [46]. Furthermore, AaERF1 and AaERF2 can attach to the GTCGAC (CBF2) and the CAACA (RAA) motifs [25]. There may also be some unknown motifs that serve as binding domains for AP2/ERF transcription factors. ChIP-Seq analysis suggests that additional binding motifs for MaDREB2 beyond (A/G)CC(G/C)AC, CCAAT(C/A)AC(A/G)A and (G/T)G(G/A/C)(A/T/C)CC(C/A)(A/G)C(A/T) in the genome of bananas [47]. This study employed dual luciferase assay, which demonstrated that VviERF003 could act on the 256 bp upstream of the *VviGT14* start codon. Additionally, a Y1H assay investigated the interaction between VviERF003 and the 127 bp upstream of the *VviGT14* start codon (Fig. 3). No known motifs were detected within the 256 bp range preceding the *VviGT14* promoter. Based on these findings, it is hypothesized that VviERF003 binds to the *VviGT14* promoter via an unknown motif.

ERF transcription factors have been shown to react to stress and plant hormones, including ethylene. Tomato *SIERF.F12* is linked to fruit ripening and impaired by ethylene [48]. In apple, the expression of *MdERF3* rises when treated with ethephon [43]. Similarly, the expressions of citrus *CsERF061* and pear *PpERF9* are elevated in response to ethylene [49, 50]. The analysis of grape transcriptomic data showed that the expression of ERF6-type transcription factors is positively correlated with the genes involved in ethylene and terpenoid biosynthesis [51]. This study suggests that the expressions *VviERF003* and *VviGT14,* along with the production of glycosylated monoterpenoids, respond to ethylene induction (Fig. 7ad–). Previous research indicates a link between ethylene and terpenoid production, as demonstrated by an increase in (+)-valencene concentration following exogenous ethylene treatment in sweet oranges and a decrease following 1-MCP treatment [21]. Ethylene has also been shown to promote glycosylated monoterpenoid accumulation. The concentration of linalyl-*β*-*D*-glucoside rises during peach fruit ripening, in tandem with ethylene production [11, 23]. Based on these findings, we propose that *VviERF003* expression induced by ethylene positively regulates the transcription of *VviGT14,* resulting in the accumulation of glycosylated monoterpenoids. Previously, our report demonstrated that ABA promotes the expression of *VviGT14* and reduces the expression of *VviWRKY40.* This implies that there is a negative regulatory cascade among ABA, VviWRKY40, and VviGT14, ultimately resulting in the accumulation of glycosylated monoterpenoids [27]. The current investigation indicated that *VviERF003* is unresponsive to ABA induction, which suggests that the regulation of VviERF003 on *VviGT14* expression may be independent of ABA signaling (Fig. 7c and d). Notably, the expression of *VviNCED1*, which encodes a crucial enzyme in ABA biosynthesis in grapes and accumulation of ABA, was enhanced and suppressed by ethephon and 1-MCP, respectively (Fig. 7cf–). This indicates that ethylene may stimulate the accumulation of glycosylated monoterpenoids through the regulation of *VviNCED1* expression, causing an increase in ABA concentration. Subsequently, this inhibits the inhibition of *VviGT14* expression by VviWRKY40.

In grape berries, a transient increase of endogenous ethylene production occurs before véraison and then a gradual decrease occurs after véraison while the level of ABA increases sharply as véraison approaches and declines gradually during ripening [52, 53]. Additionally, monoterpenoid accumulation shows a pattern of change with the ripening process (Fig. 1c). According to our results, elevated ethylene levels prior to véraison promote the accumulation of ABA, which in turn promotes *VviERF003* and represses *VviWRKY40* expression, thereby increasing *VviGT14* expression and the accumulation of glycosylated monoterpenoids at véraison [27], which is in line with previously reported results that the levels of glycosylated monoterpenoids are relatively low in the early stages of berries’ development and begin to increase rapidly from véraison, reaching a high level at maturity [4]. Our results illuminated a potential molecular mechanism of ethylene in influencing glycosylated monoterpenoids accumulation during grape development and provided a perspective for the regulation of glycosylated monoterpenoids accumulation via ethylene.

## Conclusion

**Figure 8:**
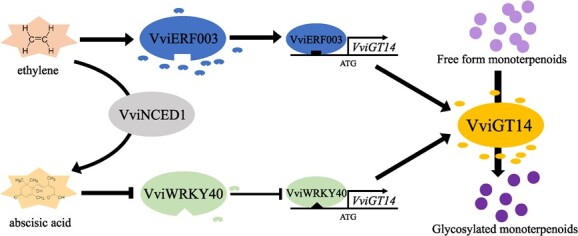
A model showing the underlying mechanism of VviERF003 and VviWRKY40 regulating the synthesis of glycosylated monoterpenoids via targeting *VviGT14* in grapes.

This study presents a mechanism for the positive regulation of glycosylated monoterpenoid biosynthesis facilitated by VviERF003. Specifically, VviERF003 is induced by ethylene, resulting in an upregulation of *VviGT14* expression, leading to a production of glycosylated monoterpenoids. In contrast, VviERF003 is not induced by ABA, but ethylene stimulates *VviNCED1* expression and leads to ABA synthesis. Building on our previous research [27], VviWRKY40 exhibits a negative response to ABA induction, resulting in weakened inhibition of *VviGT14* expression and subsequently promoting glycosylated monoterpenoid synthesis (Fig. 8). These findings shed light on a novel regulatory mechanism involving ethylene, VviERF003 and glycosylated monoterpenoid synthesis in grape berry development.

## Materials and methods

### Plant materials

For the purpose of spatiotemporal expression analyses of particular genes, samples of grape berries, leaves (10-day-old), flowers, stems, and tendrils were obtained from ‘Muscat blanc à Petit grain’ grapevines at the China Agricultural University Zhuozhou Experimental station in Hebei, China. The grape berries were collected at 14, 28, 56, 70, 84, 98, and 112 days after bloom (DAB).

For hormone treatments, ‘Muscat blanc à Petit’ grapes were collected at 0–7 days before véraison (E-L 35), and ‘Jingxiangyu’ grapes were collected during véraison from the greenhouse of the Yanhuai Valley Grape and Wine Industry Federation located in Yanqing, Beijing, China.

For the gene overexpression experiment, ‘Cabernet Sauvignon’ calli were obtained following our previous reports [54, 55]. The calli underwent culture on B5 solid medium, equipped with 3.21 g/L B5 basic medium, 30 g/L sucrose, 2.5 g/L acid-hydrolyzed casein, 0.2 mg/L kinetin, 0.1 mg/L 1-naphthyl acetic acid, and 3.0 g/L plant gel at pH 5.8–6.0 and kept in the dark at 25°C. These calli were subcultured at 25-day intervals.

Tobacco (*Nicotiana benthamiana*; 7 weeks old), *A. thaliana* (ecotype Columbia; 4 weeks old), and tomato (*Solanum lycopersicum* L. cv Micro Tom) seedlings were grown in soil (mixture of substrate, peat, and vermiculite 3:1:1) in a climate chamber with a light/dark photoperiod of 16/8 h and kept at a temperature of 23 ± 1°C. These plants were used in the following experiments.

### Hormone treatments

Grapes were fully immersed in solutions of 1000 mg/L ethephon, 1000 mg/L ABA, and 1.0 mmol/L ABA biosynthesis inhibitor NDGA, respectively, for 15 min in sealed containers. The control group was treated with distilled water. The grapes were subsequently air-dried until the water stains on the surface disappeared and then transferred to an incubator set to a 16/8 h light/dark photoperiod at 23 ± 1°C.

Regarding the treatment with the inhibitor of ethylene reception 1-MCP, the grapes were immersed in distilled water for 15 min, left to air dry, and subsequently placed in a sealed container with 1 μL/L 1-MCP and cultured in a 16/8 h light/dark photoperiod at 23 ± 1°C. 1-MCP (1 μL/L) gas was produced by placing 5 mL of 0.0384 g/mL 1-MCP (content ≥3.5%) solution in the 3 L sealed container. The aforementioned samples, which received treatments of treated with ethephon, ABA, and corresponding inhibitors, were taken from the incubator at 0, 2, 4, 6, 12, and 24 h. Subsequently, they underwent freezing in liquid nitrogen and were stored in a refrigerator at −80°C, in order to analyse their gene expression and concentrations of monoterpenoids.

### Gene isolation, phylogenetic tree construction, and multiple sequence alignment

The full-length coding sequence (CDS) of *VviERF003* was obtained by cloning from cDNA generated through reverse transcription of grape RNA from ‘Muscat blanc à Petit grain’. The utilized primers can be found in Table S5 (see online supplementary material). The conserved domains were analysed by referencing the NCBI-CCD database (https://www.ncbi.nlm.nih.gov/Structure/cdd/wrpsb.cgi). Additionally, a collection of AP2/ERF protein sequences from various species were downloaded from NCBI. The ClustalX version 2 software was used for amino acid sequence alignment, with homologous regions depicted through the BoxShade online website (http://www.ch.embnet.org/software/BOX_form.html). For phylogenetic analysis, MEGA version 11 software was employed to construct a phylogenetic tree that included VviERF003 and other members of the AP2/ERF family, using the neighbor-joining method and 1000 bootstrap replicates.

### Subcellular localization of VviERF003

The CDS of *VviERF003* lacking the stop codon was inserted upstream of the green fluorescent protein (GFP) reporter gene in the pEZS-NL plasmid. Additionally, AtFBI1-mCherry, a nucleus-localized protein, was also introduced into the same plasmid. The resulting recombinant plasmids were transformed into *Arabidopsis* leaf protoplasts using a modified polyethylene glycol technique outlined by Li *et al.* [27]. Subsequently, fluorescence was observed under a co-focal scanning microscopy (Zeiss LSM780, Oberkochen, German). The excitation wavelengths used for the detection of GFP and mCherry were 488 nm and 587 nm, respectively. Table S5 (see online supplementary material) lists the used primers. The transient expression assay was repeated at least three times.

### Yeast one-hybrid assay

The assay of Yeast One-Hybrid (Y1H) was performed using the Matchmaker Gold Yeast One-Hybrid Library Screening System (Clontech, Mountain View, CA, USA). The *VviGT14* promoter fragments truncated to 127 bp upstream of the start codon were cloned into the pAbAi vectors to construct the bait plasmids. The CDS of *VviERF003* was introduced into pGADT7 to obtain the prey plasmids. Bait plasmids were linearized and transformed into Y1HGold as bait strains, while prey plasmids and the empty vector pGADT7 were also transformed into bait strains. The yeast cells that underwent transformation were spread onto solid synthetic dropout medium without leucine (Leu) in the presence or absence of AbA. The growth of cells on both types of media confirmed the interactions between VviERF003 and *VviGT14* promoter fragments. Table S5 (see online supplementary material) displays the primers used in the process, while further details can be found in Wei *et al.*’s previous publication [54].

### Dual-luciferase transient expression assay

To examine the transcriptional activity of VviERF003, a reporter vector was created with the Firefly luciferase (LUC) gene sequence and five GAL4 AD copies upstream of the minimal CaMV35S promoter. Additionally, another reporter vector carrying the Renilla luciferase (REN) gene, driven by the CaMV35S promoter, served as an internal control. To generate an effector vector, the CDS of *VviERF003* was inserted into the pRT-BD (pBD) vector upstream of the GAL4 DNA-binding domain. The pBD without genetic material was employed as the negative control, while the pBD harboring the VP16 activation domain was used as the positive control. The plasmids carrying the reporter and effector components were introduced into *Arabidopsis* leaf protoplasts by following the established protocol [27, 56].

To evaluate the binding capability of VviERF003 with distinct *VviGT14* promoter fragments, the effector vector was created by integrating the CDS of *VviERF003* into pCAMBIA1301. The various fragments of the *VviGT14* promoter were cloned into the pGreenII 0800-LUC vector to create the reporter vectors. The pCAMBIA1301 vector was used as a control. *Agrobacterium tumefaciens* strain EHA105 was utilized to co-transform the constructed effector and reporter plasmids into tobacco leaves, following previously established protocols [55].

The Dual-Luciferase® Reporter Assay System (Promega, Madison, WI, USA) was then used to measure luciferase activities of both LUC and REN. For each assay, we conducted three independent experiments, each with a minimum of six replicates. Please refer to Table S5 (see online supplementary material) for the primers used in these experiments.

### Stable transformation assay of *VviERF003* in grape calli and tomato

*A. tumefaciens* strain GV3101, which contained the pCXSN-VviERF003 recombinant plasmid, was used to stably overexpress *VviERF003* using our previously described methodology [55]. The calli and the constructed *A. tumefaciens* strain GV3101 were co-incubated in the dark for 3 days after being shaken gently together for 10 min. Afterwards, the transformed calli were rinsed with sterile water, alternately treated with cefalexin and carboxymycin, and then cultured on the solid B5 media containing hygromycin until new calli had developed.

Seven-day-old Mirco Tom tomato cotyledon explants were infected with *A. tumefaciens* and cultured on T1 medium [Murashige and Skoog medium (MS) 4.4 g/L, sucrose 15 g/L, 6-benzylaminopurine 1.0 mg/L, indole-3-acetic acid (IAA) 1.0 mg/L] for 48 h in the dark. The cotyledon explants were then sequentially transferred to T21, T22, and Tr solid mediums to induce the generation of shoot and roots. The composition of T21 medium includes 4.4 g/L of MS, 15 g/L of sucrose, 1.0 mg/L of zeatin (ZT), 0.1 mg/L of IAA, 10 mg/L of hygromycin B (Hyg), and 200 mg/L of timentin (Ti). T22 medium is similar to T21, but with 0.5 mg/L of ZT, 1.0 mg/L of gibberellin, 10 mg/L of Hyg, and 300 mg/L of Ti. Tr medium contains 2.2 g/L of MS, 7.5 g/L of sucrose, 2.0 mg/L of indole-3-butyric acid, 5 mg/L of Hyg, and 150 mg/L of Ti. After the roots had formed, the seedlings were transplanted into the soil. The fruits were harvested 47 days after flowering.

### Transient transformation assay of *VviERF003* in calli, leaves, and berries of grapes

To construct *VviERF003* silencing vector, a 225 bp fragment with its reserve sequence of *VviERF003* was cloned into the pNC-Cam1304-RNAi vector on either side of an intron. The *A. tumefaciens* strain GV3101, carrying recombinant plasmids, was combined with grape calli under a vacuum of −0.8 MPa for 10 min. Following rapid pressurization, the calli drained of surface moisture were cultured in the dark for 3 days.

For transient overexpression in grape leaves, the recombinant pCAMBIA1301-VviERF003 construct was transformed into *A. tumefaciens* strain EHA105. The leaves of ‘Yeniang-2’ were fully immersed in the suspension of *A. tumefaciens* with recombined vectors and vacuum infiltrated for 30 min at −0.8 MPa. Following three days of incubation while wrapped in moist gauze in a dark environment, the leaves were harvested for further analysis [55]. Table S5 (see online supplementary material) displays the primers used in the process.

To conduct transient overexpression and silencing experiments in grape berries, we followed a specific protocol. First, we immersed mature ‘Summer Black’ berries in an *A. tumefaciens* suspension and vacuumed them for 15 min at −0.8 MPa. Next, we dried the berries with absorbent paper and placed them in a petri dish covered with moist gauze. We then incubated the berries for three days in a 16/8 hour light/dark photoperiod at 23 ± 1°C. In wine grape, monoterpenoids are mainly concentrated in the skin [57, 58], we peeled off the berry skins for further analysis. The experiment utilized *A. tumefaciens* strains GV3101 carrying pCAMBIA1301-VviERF003 and pNC-Cam1304-RNAi-VviERF003 mentioned above for *VviERF003* overexpression and silencing, respectively.

### Detection of free and glycosylated terpenoids

Free and glycosylated terpenoids were extracted according to a modified version of the previous method [59]. Grape calli, leaves, or berries were mixed with polyvinylpolypyrrolidone and ground into a powder using liquid nitrogen. To extract free-form terpenoids, 4 g of callus powder was added to a vial containing 3 mL of citrate buffer (0.2 mol/L, pH = 5.0) and 1.5 g of NaCl. The vials were tightly sealed and macerated at 4°C for 16 h. To extract the total terpenoids, callus powder of the same weight was incubated with 3 mL citrate buffer (0.2 mol/L, pH = 5.0) and 100 g/L of glycosidase AR 2000 (Creative Enzymes, NY, USA) in a vial at 40°C for 16 h. Before testing, 10 μL of linalool-d3 (0.01 mg/mL) was added as an internal standard to each vial. Due to the low concentration of glycosylated terpenoids in grape calli, we calculated the glycosylated terpenoid concentration by subtracting the free form from the corresponding total concentration.

To prepare the leaves and grape skin, we macerated 5 g of the powder in 25 mL of citrate buffer (0.2 mol/L, pH = 5.0) at 4°C for 16 h in the dark. Similarly, 17 g of the tomato powder was mixed with 25 mL of citrate buffer (0.2 mol/L, pH = 5.0). For grape berries, we directly melted the powder for 16 h at 4°C, and then filtered the juice of each material with six layers of gauze to obtain a clear solution. Five milliters of juice, along with 1.5 g of NaCl, were added to a vial to detect free-form terpenoids. To extract the glycosylated terpenoids, an additional 2 mL of clear juice for leaves, grape skin or tomato or 1 mL for berries was filtered using Cleanert PEP-SPE resin (Bonna-agela Technologies, China, 200 mg/6 mL), following the previously described method [15]. The extract was evaporated until dryness, then dissolved in 5 mL of citrate buffer (0.2 mol/L, pH = 5.0). Following that, it was incubated with 100 g/L glycosidase AR 2000 (Creative Enzymes, NY, USA) at 40°C for 16 h. To prepare for testing, an internal standard of 10 μL of 4-methyl-2-pentanol (1.008 g/L for leaves, berries, and skin, 0.1 g/L for tomato) was added to each vial.

The terpenoids were then extracted via headspace-solid phase microextraction (HS-SPME) with a 2 cm DVB/CAR/PDMS 50/30 μm SPME fiber (Supelco, Bellefonte, PA, USA) at 40°C for 30 min with stirring. The volatile compounds were analysed using an Agilent 6890 gas chromatograph coupled with an Agilent 5975C mass spectrometer (Agilent Technologies, Santa Clara, CA, USA). Separation of the volatiles was achieved with an HP-INNOWAX capillary column (60 m × 0.25 mm × 0.25 μm, J &W Scientific, Folsom, CA, USA), while the instrument temperature was set according to the previously established method [4]. Terpenoids was identified using reference standards or the standard NIST11 library, when standards were not available. Quantification was performed using the internal standard.

### Extraction and determination of ABA

Seeded grape berries were ground to a powder in a liquid nitrogen environment. A total of 0.5 g grape powder was mixed with 2.5 mL 50% cold acetonitrile (ACN) solution and 10 μL internal standard solution (0.1 mg/L triphenyl phosphate (TPP) solution) and then sonicated for 15 min in an ice water bath. The clarified supernatant was then collected and the above steps were repeated after adding a further 2.5 ml of ACN solution to the precipitate. The extracts were collected into an Oasis HLB column (Waters, Milford, MA, USA, 3 cc, 60 mg) by a high-throughput automatic solid-phase extractor (Fotector-08HT, Raykol, Xiamen, China) and eluted with 3 mL CH_2_Cl_2_ containing 0.5% formic acid and 2.5% methanol (MeOH), the collection procedure being described in our previous report [60]. The eluent was dried under a gentle nitrogen flow and re-solubilized with 200 μl MeOH.

The final extracts were detected on a 1260 Infinity II UHPLC instrument coupled with a 6470B MS/MS system equipped with an Agilent jet stream electrospray ionization source (AJS-ESI, Agilent, Santa Clara, CA, USA). The separation of ABA was performed on a Zorbax Eclipse Plus C18 column (95 A, 1.8 mm, 2.1 × 50 mm, Agilent, Santa Clara, CA, USA). ABA were monitored by multiple reaction monitoring in a fast-switching negative mode of electrospray ionization. The ion source conditions were set according to Yao *et al.* [60]. ABA was quantified by ratio of ABA to the peak area of the internal standard TPP and an external standard curve.

### RNA extraction, cDNA synthesis, and quantitative real-time PCR analysis

Total RNA was extracted from grape berries, grape calli, leaves, flowers, stems, and tendrils using the Spectrum™ Plant Total RNA Kit (Sigma-Aldrich, St Louis, MO, USA) or the Universal Plant Total RNA Extraction Kit (BioTeke, China) following the manufacturer’s instructions. The concentration and quality were then detected using microspectrophotometry and agarose gel electrophoresis. The process of reverse transcription of RNA into cDNA was performed using the HiScript ® IIQ RT SuperMix for qPCR (+gDNA wiper) (Vazyme, Nanjing, China). The CFX96/384 Touch Real-Time PCR Detection System (Bio-Rad, CA, USA) was utilized to perform quantitative real-time PCR with the ChamQ Universal SYBR qPCR Master Mix (Vazyme, Nanjing, China). The reactions for each sample were carried out in triplicate. Table S5 (see online supplementary material) lists the primers used in this study.

### Statistical analysis

All experimental procedures in this study were conducted in triplicates. Graphs were generated using GraphPad prism 8.3.0 software (GraphPad Software, Boston, MA, USA). Significance analysis was carried out using Student’s *t*-test with a 95.0% confidence level (*, P < 0.05) or 99.0% confidence level (**, P < 0.01).

### Accession numbers

Sequences from this article can be queried from NCBI data libraries (NCBI, https://www.ncbi.nlm.nih.gov/) under the following accession numbers: *VviERF003* (XM_002285337.3), *VviGT14* (XM_002285734.2), *VviACO1* (XM_002273394.3), *VviETR2* (XM_002284471.3), *VviNCED1* (XM_019216859.1).

## Acknowledgements

This work was supported by the National Natural Science Foundation of China (No. U20A2042 to C.-Q.D. and 31772278 to Q.-H.P.).

## Author contributions

Q.-H.P. and C.-Q.D. contributed to the conception and design of this study. Y.-C.W. performed the majority of the experiments and data analysis. X.-Y.L. performed the subcellular localization and transcriptional activity assay. Y.W., H.-M.Z., and X.M. participated in the transgenic calli experiment and the measurement of terpenoids. Y.-C.W. wrote the manuscript. Q.-H.P., Y.W., and Y.-C.W. reviewed and revised the manuscript. All authors have read and approved the final version of the manuscript.

## Data availability

All study data are presented in the submitted article.

## Conflict of interest statement

The authors declare no competing interests.

## Supplementary data

Supplementary data is available at *Horticulture Research* online.

## Supplementary Material

Web_Material_uhae065
