# Supplementary material for: Ethylene-responsive VviERF003 modulates glycosylated monoterpenoid synthesis by upregulating VviGT14 in grapes
Source: Hortic Res. 2024 Feb 28;11(4):uhae065. doi: 10.1093/hr/uhae065 (PMC11059816; doi:10.1093/hr/uhae065)
Supplement: Web_Material_uhae065 [file web_material_uhae065.zip › Supplementary information.docx]

**Supplemental data**


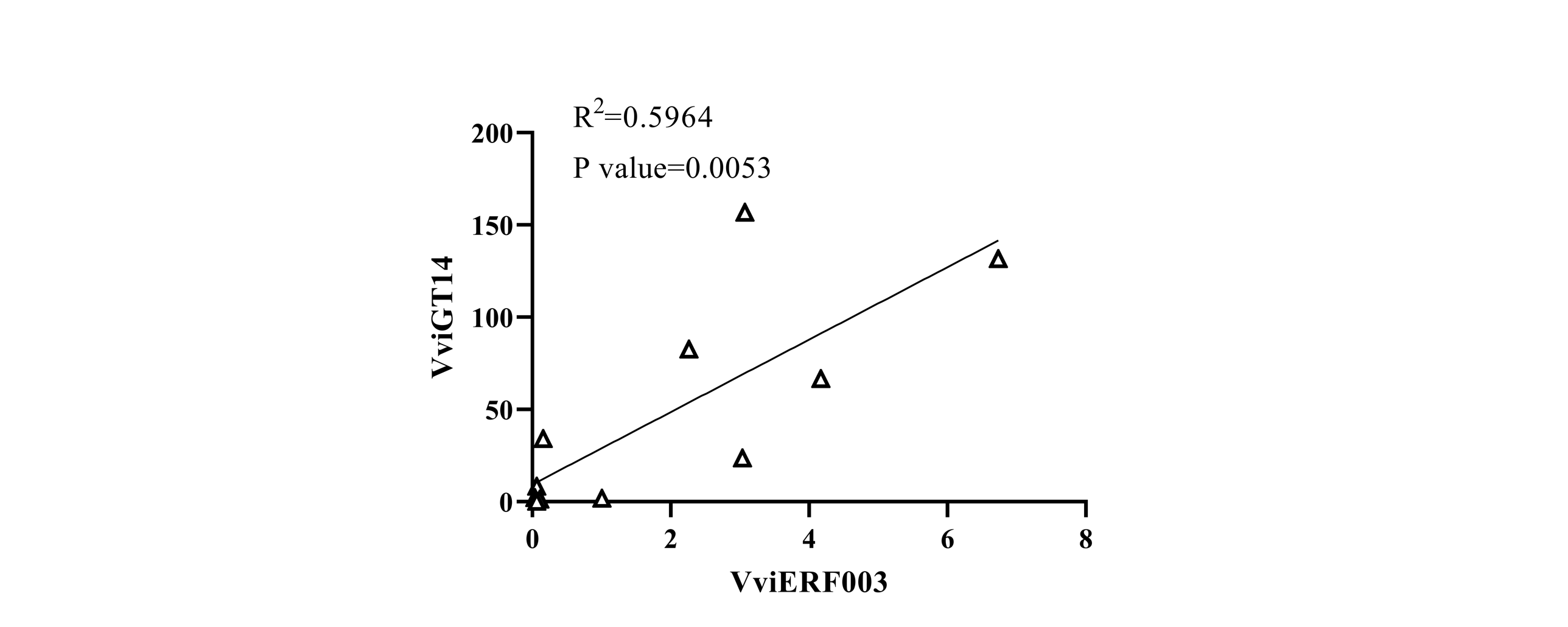


Supplementary figure S1. Correlation analysis of *VviGT14* and *VviERF003* expression levels.





Supplementary figure S2. Analysis of transient-acting elements on the *VviGT14* promoter.





Supplementary figure S3. Transient expression of *VviERF003* in ‘Yeniang-2’ grape leaves. **a** The relative expressions of *VqGTs* in transgenic leaves. **b** Concentrations of glycosylated and **c** free form monoterpenoids relative in transgenic leaves.





Supplementary figure S4. Effects of transient expression of *VviERF003* in ‘Summer Black’ berry skins. Effects of **a** *VviERF003* overexpression and **b** VviERF003 interfered expression on the concentrations of glycosylated monoterpenoids in grape skins. Asterisk indicates statistical significance using independent sample t-test, *, p < 0.05; **, p < 0.01.





Supplementary figure S5. Contents of total or free-form monoterpenoids in grape calli overexpressing *VviERF003* and interfering VviERF003. **a** Concentrations of total monoterpenoids after enzymatic hydrolysis and **b** concentrations of free form monoterpenoid compounds in WT and *VviERF003* overexpressing calli. **c** Concentrations of free-form monoterpenoid compounds in calli in which *VviERF003* expression is transiently interfered. Asterisk indicates statistical significance using independent sample t-test, *, p < 0.05.





Supplementary figure S6. Relative expression of related genes and concentrations of monoterpenoids in grape berries upon ethephon treatment. **a** The relative expression of *VviACO1* and *VviETR2* and **b** the contents of glycosylated and **c** free form monoterpenoids in ‘Muscat Blanc’ berries before véraison under ethephon treatment. Asterisk indicates statistical significance using independent sample t-test, *, p < 0.05.


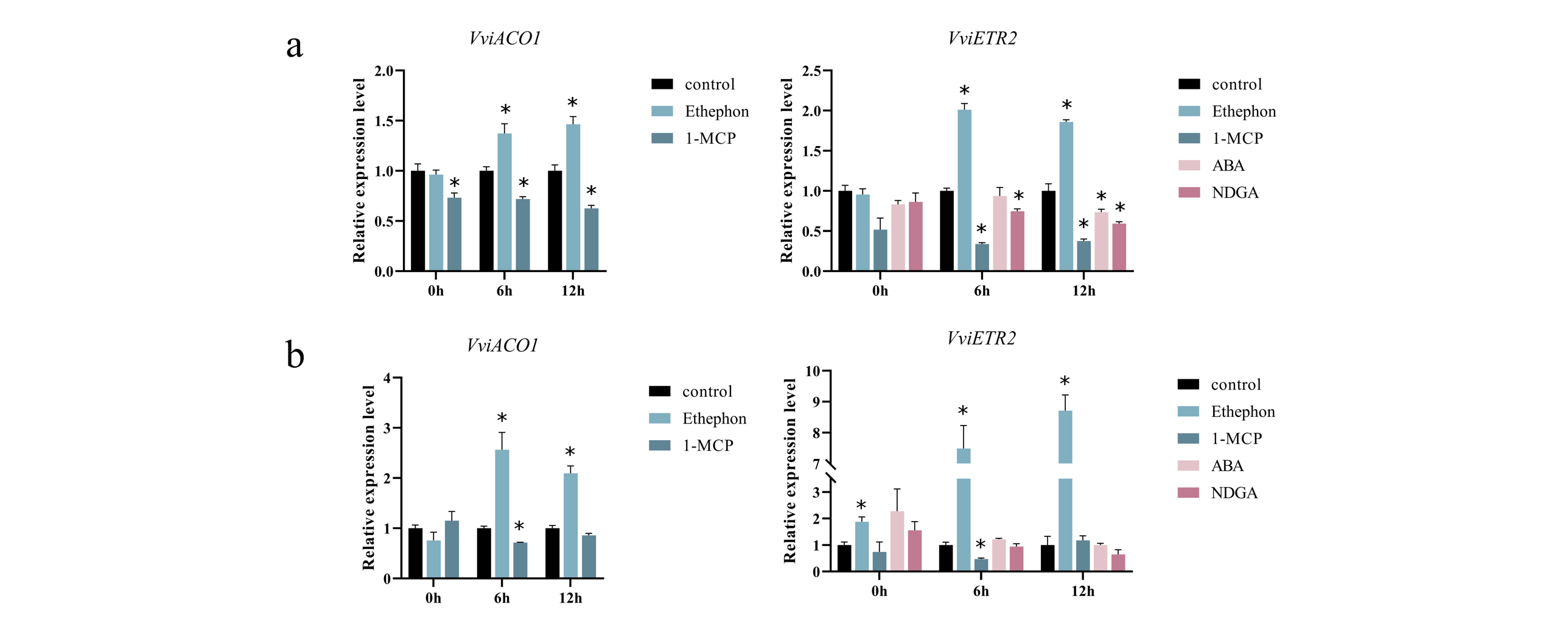


Supplementary figure S7. Relative expression of related genes in grape berries after hormone treatment. **a** The relative expression of *VviACO1* and *VviETR2* in ‘Jingxaingyu’ and **b** ‘Muscat Blanc’ berries at véraison. Asterisk indicates statistical significance using independent sample t-test, *, p < 0.05.

Table S1. Primers used in the study.

| **Function** | **Primer name** | | **Primer sequence(5’→3’)** |
| --- | --- | --- | --- |
| Full-length gene cloning | ERF3-F | ATGGCCAGACCTCAGCAGCGATATCG | |
|  | ERF3-R | CTACATTATGGAGCCTGAGCAGAGCT | |
|  | SlActin-F | ATGGCTGACGGTGAGGATAT | |
|  | SlActin-F | TTAGAAACACTTCCTGTGGACAATG | |
| Yeast one-hybrid assay | AD-F | TATGGCCATGGAGGCCAGTGAATTCATGGCCAGACCTCAGCAGCG | |
|  | AD-R | TCTGCAGCTCGAGCTCGATGGATCCCTACATTATGGAGCCTGAGCAGAGCT | |
|  | ABAP7-F | TACATACAGAGCACATGCCTCGAGATTAAATCAGTATTATCAATGAT | |
|  | ABAP7-R | AAGCTTGAATTCGAGCTCGGTACTTTTGCTTTGGGATGCCCT | |
| Overexpression construct | pcxsn-F | TTACGAACGATACTCGAGGGGGATCATGGCCAGACCTCAGCAGCG | |
|  | pcxsn-R | GATCGGGGAAATTCGCTAGTGGATCCTACATTATGGAGCCTGAGCAGAGCT | |
|  | 1300-F | AGAACACGGGGGACTCTAGAGGATCATGGCCAGACCTCAGCAGCG | |
|  | 1300-R | TTGAACGATCGGGGAAATTCGAGCTCTACATTATGGAGCCTGAGCAGAGCT | |
| Silent construct | RNAiE3-F | AGTGGTCTCTGTCCAGTCCTCTTCTTTCAGCTACTTTAACTGCC | |
|  | RNAiE3-R | GGTCTCAGCAGACCACAAGTCACTTGAACTTTCTTCACAACCC | |
| Subcellular localization | NLS-F | AGAGGACACGCTCGAATGGCCAGACCTCAGCAGC | |
|  | NLS-R | CCGCGGTACCGTCGACATTATGGAGCCTGAGCAGAGC | |
| Dual luciferase assay | 0800P1-F | TAAGCTTGATATCGAATTCCTGCAGTTGATGGATAATGATGGGTAA | |
|  | 0800P2-F | TAAGCTTGATATCGAATTCCTGCAGCCAGTAAAAAAATTACCAACACTTT | |
|  | 0800P3-F | TAAGCTTGATATCGAATTCCTGCAGAATTCATGGACGATAACTTAGTCC | |
|  | 0800P4-F | TAAGCTTGATATCGAATTCCTGCAGGGTAAATTCATTATTCTCAACGGAAGA | |
|  | 0800P5-F | TAAGCTTGATATCGAATTCCTGCAGACTGTGATTTGAAGATAAAAAGTGTAGG | |
|  | 0800P6-F | TAAGCTTGATATCGAATTCCTGCAGGTTAAATCGATTTGGCACATGC | |
|  | 0800P7-F | TAAGCTTGATATCGAATTCCTGCAGGATTAAATCAGTATTATCAATGAT | |
|  | 0800P-R | CGGCCGCTCTAGAACTAGTGGATCCTTTTGCTTTGGGATGCCCT | |
| Transcriptional activation assay | VviERF003-BD-F | GACAGTAGCTTCATGATGGCCAGACCTCAGCAGC | |
|  | VviERF003-BD-R | GTATCGCCGTCTAGCATTATGGAGCCTGAGCAGAGC | |
| qRT-PCR analysis | VviUbiquitin-F/VqUbiquitin-F | GTGGTATTATTGAGCCATCCTT | |
|  | VviUbiquitin-R/VqUbiquitin-R | AACCTCCAATCCAGTCATCTAC | |
|  | VviGT14-R/VqGT14-R | ACCATGGAGTGGAAGCATAGGG | |
|  | VviGT14-F/VqGT14-F | TGGAAACAAGGCAGGAAAGGTG | |
|  | VqGT7-R | TACCTTGGGAACACATACTCCACTC | |
|  | VqGT7-F | TGGTGATGGATACAAGACAACACTC | |
|  | VviGT15-R | CAACACTGGAAAGCATCTCCGAAGG | |
|  | VviGT15-F | ATCACTCTCCAACTCCAATCCCACC | |
|  | VviERF003-F | CGCCACCCACTTCTGAAGAC | |
|  | VviERF003-R | GCTTGGAAGACGACGACTGG | |
|  | VviNCED1-F | TTCCACGGCACCTTCATAAGC | |
|  | VviNCED1-R | ATATGCGGACCATCCCTCTGG | |
|  | VviNCED2-R | TCGTCGATCACAATCACGGC | |
|  | VviNCED2-F | AGGGCTTGATTCGCACTTGG | |
|  | VviACO1-F | GCCACAGTTTGAGTCCTTTGC | |
|  | VviACO1-R | AGCAGCAGGCATGGTACATC | |
|  | VviACS2-F | GAACCTGGATGGTTCCGCTG | |
|  | VviACS2-R | CCTGCAGGTGATTTGAGTATCCAG | |
|  | VviACS6-F | GACGTATCAGCCGTTGGATGG | |
|  | VviACS6-R | TCAGCCAAGCAGAAGCAGAG | |
|  | VviETR2-F | GCAGAAGGCTAGCTCAGTTGATG | |
|  | VviERT2-R | AGAGAGTTGGAATGTGGGTGC | |

Table S2. Concentrations (μg/g, mean, n = 3) of glycosylated monoterpenoids in developing grape berries.

| **Cluster** | **Substrate** | **Days after Bloom** | | | | | | |
| --- | --- | --- | --- | --- | --- | --- | --- | --- |
|  |  | **14** | **28** | **56(véraison)** | **70** | **84** | **98** | **112** |
| 1 | Citronellol | 0.5923±0.0178 | 0.1078±0.0195 | 0.0252±0.0008 | 0.0343±0.0049 | 0.047±0.0053 | 0.02±0.0059 | 0.0355±0.0043 |
|  | Nerol | 0.8746±0.1214 | 0.2831±0.0603 | 0.0922±0.0156 | 0.343±0.0192 | 0.4396±0.0779 | 0.3074±0.0653 | 0.317±0.0463 |
|  | Geraniol | 1.5604±0.2399 | 0.3845±0.0733 | 0.107±0.0149 | 0.1255±0.0086 | 0.2517±0.0438 | 0.2139±0.0337 | 0.2391±0.0313 |
|  | *α*-Cyclogeraniol | 0.2328±0.0087 | 0.1409±0.0265 | 0.0963±0.0052 | 0.059±0.0071 | 0.0753±0.0055 | 0.104±0.0083 | 0.0657±0.0058 |
| 2 | Linalool | 0.0882±0.0131 | 0.0292±0.005 | 0.0045±0.0012 | 0.0471±0.0042 | 0.1166±0.0081 | 0.1833±0.0295 | 0.1756±0.0211 |
|  | D-Limonene | 0.2953±0.0047 | 0.0633±0.0071 | 0.0364±0.0005 | 0.0746±0.006 | 0.1043±0.0084 | 0.1896±0.0097 | 0.1176±0.0065 |
|  | *β*-Citral | 0.0193±0.0004 | 0.0052±0.0008 | 0.0035±0.0002 | 0.0085±0.0014 | 0.012±0.0004 | 0.0215±0.0016 | 0.0145±0.0018 |
|  | *α*-Citral | 0.034±0.0009 | 0.0088±0.0013 | 0.0054±0.0001 | 0.0103±0.0016 | 0.0158±0.0006 | 0.028±0.0015 | 0.0211±0.0026 |
| 3 | *γ*-Terpinene | 0.377±0.0014 | 0.0561±0.0006 | 0.0575±0.0001 | 0.0639±0.0063 | 0.0645±0.0003 | 0.2838±0.0012 | 0.106±0.0011 |
|  | Terpinolene | 0.3918±0.0017 | 0.0617±0.002 | 0.059±0.0002 | 0.0668±0.0016 | 0.0756±0.002 | 0.2923±0.0026 | 0.116±0.0017 |
|  | trans-Rose oxide | 0.0264±0 | 0.004±0 | 0.0042±0 | 0.0042±0 | 0.0044±0 | 0.0203±0 | 0.0074±0 |
|  | (*E*,*Z*)-allo-Ocimene | 0.3994±0.0021 | 0.0619±0.0017 | 0.0587±0.0002 | 0.0616±0.0008 | 0.0703±0.0013 | 0.2889±0.0019 | 0.1134±0.0015 |
|  | Neo-allo-ocimene | 0.3986±0.0042 | 0.0621±0.0022 | 0.0586±0.0004 | 0.0622±0.0012 | 0.0716±0.0015 | 0.2893±0.0023 | 0.1146±0.0016 |
|  | 2,6-Dimethyl-1,3,5,7-octatetraene, E,E- | 0.3653±0 | 0.0534±0.0001 | 0.0567±0 | 0.0562±0.0001 | 0.0579±0.0001 | 0.2778±0.0001 | 0.0996±0.0001 |
|  | cis-linalool oxide(F) | 0.028±0 | 0.0046±0.0002 | 0.0045±0 | 0.0042±0 | 0.0042±0 | 0.0201±0 | 0.0072±0 |
|  | Terpinen-4-ol | 0.059±0.0001 | 0.0089±0.0002 | 0.0091±0 | 0.0092±0.0002 | 0.0097±0.0001 | 0.0441±0.0001 | 0.0162±0 |
|  | p-Menth-1-en-9-al | 0.0166±0.0004 | 0.0034±0.0003 | 0.0031±0.0001 | 0.0028±0.0001 | 0.0038±0.0001 | 0.0122±0.0002 | 0.0057±0.0003 |
|  | (±)-Menthol | 0.059±0.0009 | 0.0085±0.0001 | 0.009±0 | 0.0089±0.0001 | 0.0091±0.0001 | 0.0443±0.0006 | 0.0166±0.0013 |
|  | 1-Octanol, 3,7-dimethyl- | 0.0626±0.0024 | 0.0197±0.0097 | 0.0148±0.0049 | 0.0135±0.0005 | 0.0129±0.0027 | 0.0546±0.0007 | 0.0266±0.0088 |
|  | *α*-Terpineol | 0.5005±0.0129 | 0.1024±0.0089 | 0.0722±0.0015 | 0.0884±0.0043 | 0.1117±0.0067 | 0.3194±0.0097 | 0.1557±0.007 |
|  | *α*-Terpinene | 0.4047±0.0041 | 0.0619±0.0034 | 0.06±0.0005 | 0.0695±0.0015 | 0.0816±0.0026 | 0.2982±0.0035 | 0.1229±0.0025 |
|  | *β*-Phellandrene | 0.6118±0.003 | 0.093±0.0035 | 0.091±0.0004 | 0.0999±0.0011 | 0.1126±0.0009 | 0.4453±0.0035 | 0.1761±0.0014 |
|  | trans-*β*-Ocimene | 0.4416±0.004 | 0.0727±0.0035 | 0.0614±0.0004 | 0.0692±0.0019 | 0.089±0.0034 | 0.3083±0.0048 | 0.1367±0.0038 |
|  | p-Cymene | 0.0819±0.0002 | 0.0123±0.0002 | 0.0125±0 | 0.0134±0.0003 | 0.0144±0.0002 | 0.0622±0.0004 | 0.0234±0.0001 |
|  | Rose oxide | 0.027±0.0001 | 0.0046±0.0001 | 0.0044±0 | 0.0048±0.0001 | 0.0052±0.0001 | 0.0213±0.0001 | 0.0082±0.0001 |
|  | trans-linalool oxide(F) | 0.0285±0.0001 | 0.0043±0.0001 | 0.0044±0 | 0.0044±0.0001 | 0.0047±0.0001 | 0.0208±0.0002 | 0.0078±0.0001 |
|  | Nerol oxide | 0.0266±0.0001 | 0.0042±0 | 0.0044±0 | 0.0063±0.0004 | 0.0078±0.0001 | 0.0246±0.0004 | 0.012±0.0005 |
|  | Hotrienol | 0.0589±0.0003 | 0.0093±0.0002 | 0.0096±0.0002 | 0.0099±0.0002 | 0.0111±0.0001 | 0.0466±0.0006 | 0.0181±0.0006 |
|  | cis-linalool oxide(P) | 0.0267±0 | 0.0041±0 | 0.0043±0 | 0.0042±0 | 0.0043±0 | 0.0202±0 | 0.0073±0 |
|  | *β*-Myrcene | 0.8071±0.0084 | 0.1417±0.0122 | 0.1034±0.0003 | 0.1323±0.005 | 0.1716±0.007 | 0.5137±0.0095 | 0.2465±0.0104 |
|  | cis-*β*-Ocimene | 0.5835±0.0121 | 0.0994±0.0049 | 0.0713±0.0009 | 0.0993±0.004 | 0.145±0.0091 | 0.3686±0.014 | 0.1995±0.0078 |

Table S3. Concentrations (μg/g, mean, n = 3) of free form monoterpenoids in developing grape berries.

| **Cluster** | | **Substrate** | **Days after Bloom** | | | | | | |
| --- | --- | --- | --- | --- | --- | --- | --- | --- | --- |
|  |  |  | **14** | **28** | **56(véraison)** | **70** | **84** | **98** | **112** |
| 1 | *α*-Terpinene | | 0.2213±0.0036 | 0.0305±0.0014 | 0.0269±0.0008 | 0.0462±0.0028 | 0.0505±0.0009 | 0.1176±0.0004 | 0.0455±0.0002 |
|  | *β*-Phellandrene | | 0.3608±0 | 0.0384±0.0005 | 0.0357±0.0001 | 0.061±0.0004 | 0.0608±0.0022 | 0.1774±0.0002 | 0.0669±0 |
|  | Terpinolene | | 0.1966±0.0028 | 0.0377±0.0008 | 0.0298±0.0003 | 0.054±0.0037 | 0.0642±0.0019 | 0.1169±0.0002 | 0.046±0.0005 |
|  | trans-Rose oxide | | 0.0111±0 | 0.0016±0 | 0.0016±0 | 0.0017±0 | 0.0017±0 | 0.0081±0 | 0.0029±0 |
|  | (*E*,*Z*)-allo-Ocimene | | 0.2962±0.0034 | 0.0266±0.0001 | 0.0246±0 | 0.0469±0.002 | 0.0502±0.0008 | 0.1192±0.0003 | 0.046±0.0003 |
|  | Neo-allo-ocimene | | 0.2647±0.007 | 0.0258±0.0003 | 0.0245±0.0001 | 0.0438±0.0024 | 0.0483±0.002 | 0.1177±0.0004 | 0.0452±0.0006 |
|  | Terpinen-4-ol | | 0.0312±0.0006 | 0.0079±0.0012 | 0.0061±0.0002 | 0.0057±0.0006 | 0.0058±0.0001 | 0.0239±0.0103 | 0.0066±0.0001 |
|  | *γ*-Terpinene | | 0.1657±0.0011 | 0.0282±0.0004 | 0.0268±0 | 0.0303±0.0014 | 0.0327±0.001 | 0.1132±0.0003 | 0.0413±0.0001 |
|  | p-Cymene | | 0.0362±0.0003 | 0.0074±0.0002 | 0.0067±0.0001 | 0.0067±0.0002 | 0.0071±0.0001 | 0.0244±0 | 0.009±0 |
|  | Rose oxide | | 0.0127±0.0001 | 0.0019±0 | 0.0017±0 | 0.0021±0 | 0.0021±0 | 0.0083±0 | 0.0031±0 |
|  | trans-linalool oxide(F) | | 0.0149±0 | 0.0053±0.0003 | 0.0028±0.0001 | 0.0025±0.0001 | 0.0025±0 | 0.0082±0 | 0.0031±0 |
|  | 2,6-Dimethyl-1,3,5,7-octatetraene, *E,E*- | | 0.1519±0.0001 | 0.0366±0.0014 | 0.0297±0.0004 | 0.0298±0.001 | 0.031±0.0003 | 0.1114±0 | 0.0401±0.0001 |
|  | cis-linalool oxide(F) | | 0.0118±0 | 0.0033±0.0001 | 0.002±0 | 0.0022±0 | 0.0021±0 | 0.0082±0 | 0.003±0 |
|  | Nerol oxide | | 0.0112±0 | 0.0046±0.0011 | 0.0039±0.0002 | 0.0044±0.0003 | 0.0046±0.0001 | 0.0087±0.0002 | 0.0036±0 |
|  | (±)-Menthol | | 0.0234±0.0001 | 0.0035±0 | 0.0037±0 | 0.0038±0 | 0.0038±0 | 0.0181±0.0002 | 0.0064±0 |
|  | 1-Octanol, 3,7-dimethyl- | | 0.3235±0.0078 | 0.0042±0.0003 | 0.0041±0 | 0.0106±0.0056 | 0.0124±0.0005 | 0.0269±0.0015 | 0.0165±0.0007 |
|  | *β*-Citral | | 0.0118±0.0001 | 0.0011±0.0001 | 0.001±0 | 0.0014±0 | 0.0013±0 | 0.0049±0.0002 | 0.0021±0.0001 |
|  | cis-linalool oxide(P) | | 0.0126±0.0001 | 0.0022±0.0001 | 0.0017±0 | 0.0021±0 | 0.0023±0.0001 | 0.0084±0 | 0.003±0 |
| 2 | D-Limonene | | 0.3075±0.0058 | 0.039±0.0021 | 0.0229±0.0008 | 0.0981±0.0073 | 0.1153±0.0031 | 0.072±0.0005 | 0.0399±0.0011 |
|  | Linalool | | 0.411±0.0202 | 0.107±0.0054 | 0.0315±0.0026 | 0.3971±0.015 | 0.3782±0.0167 | 0.1829±0.0082 | 0.1167±0.0043 |
|  | Hotrienol | | 0.0412±0.0008 | 0.2574±0.0156 | 0.117±0.0072 | 0.1243±0.0115 | 0.1152±0.0052 | 0.026±0.0012 | 0.0165±0.0004 |
|  | *α*-Terpineol | | 0.3565±0.0122 | 0.2302±0.0216 | 0.1125±0.0086 | 0.3196±0.0325 | 0.4158±0.016 | 0.1558±0.0033 | 0.1012±0.0019 |
|  | Nerol | | 1.8058±0.1067 | 0.0213±0.0018 | 0.0057±0.0006 | 0.1168±0.0032 | 0.112±0.0035 | 0.0676±0.0042 | 0.0444±0.0026 |
|  | Geraniol | | 11.5185±0.5104 | 0.0955±0.0043 | 0.0251±0.002 | 0.3621±0.0085 | 0.3405±0.0039 | 0.249±0.0162 | 0.1383±0.0078 |
|  | *α*-Cyclogeraniol | | 0.6203±0.0089 | 0.0208±0.0013 | 0.0174±0.0009 | 0.0241±0.0023 | 0.0228±0.0006 | 0.0234±0.0004 | 0.0094±0.0003 |
|  | Citronellol | | 1.4536±0.0293 | 0.0157±0.0013 | 0.0038±0.0004 | 0.0282±0.0013 | 0.0253±0.0006 | 0.0093±0.0005 | 0.008±0 |
| 3 | cis-*β*-Ocimene | | 0.9886±0.2693 | 0.0561±0.0019 | 0.0361±0.0003 | 0.163±0.0083 | 0.1714±0.011 | 0.166±0.004 | 0.0827±0.0015 |
|  | *α*-Citral | | 0.0827±0.0012 | 0.0032±0.0004 | 0.0013±0 | 0.0058±0.0001 | 0.0053±0.0003 | 0.0089±0.0001 | 0.0054±0 |
|  | *β*-Myrcene | | 1.0589±0.0166 | 0.0579±0.0024 | 0.0426±0.0022 | 0.1688±0.0079 | 0.1748±0.002 | 0.2232±0.0015 | 0.1002±0.0002 |
|  | trans-*β*-Ocimene | | 0.4736±0.0121 | 0.0331±0.0006 | 0.027±0 | 0.0859±0.0049 | 0.092±0.0029 | 0.1328±0.0006 | 0.0566±0.0009 |
|  | p-Menth-1-en-9-al | | 0.0085±0.0001 | 0.0046±0.0003 | 0.0023±0.0002 | 0.0036±0.0003 | 0.005±0.0007 | 0.0049±0.0003 | 0.0023±0.0001 |

Table S4. Concentrations of free-form monoterpenoids in ‘Summer Black’ grape skins with VviERF003 overexpression or RNA interference. (**μg/kg**, mean, n = 3)

| **Components** | **Overexpression group** | | **RNA interference group** | |
| --- | --- | --- | --- | --- |
|  | Control | OEERE003 | Control | RNAi-ERF003 |
| Linalool | 6.65 ± 0.21 | 9.63 ± 0.26 ** | 12.7 ± 0.22 | 7.69 ± 0.11 ** |
| Citronellol | 93.31 ± 1.33 | 105.71 ± 5.87 * | 151.68 ± 5.51 | 131.59 ± 2.62 ** |
| Nerol | 45.07 ± 0.74 | 106.65 ± 13.95 ** | 132.6 ± 10.02 | 52.04 ± 2.23 ** |
| Geraniol | 28.67 ± 3.13 | 80.18 ± 6.77 ** | 122.46 ± 9.36 | 46.64 ± 0.56 ** |
| 1-Terpinenol | 3.74 ± 0.15 | 4.05 ± 0.15 | 4.3 ± 0.12 | 3.89 ± 0.03 ** |
| Terpinen-4-ol | 5.98 ± 0.11 | 9.04 ± 0.42 ** | 10 ± 0.42 | 6.23 ± 0.26 ** |
| *α*-Terpineol | 3.1 ± 0.02 | 7.5 ± 0.87 ** | 11.28 ± 0.33 | 3.96 ± 0.06 ** |
| (±)-Menthol | 4.63 ± 0.19 | 5.13 ± 0.13 * | 5.04 ± 0.4 | 4.71 ± 0.25 |
| p-Mentha-1,5-dien-8-ol | 3.22 ± 0.01 | 3.55 ± 0.09 ** | 3.77 ± 0.03 | 3.46 ± 0.01 ** |
| *β*-Myrcene | 21.33 ± 0.4 | 33.37 ± 2.58 ** | 41.68 ± 1.65 | 24.99 ± 1.26 ** |
| *α*-Terpinene | 23.44 ± 0.19 | 27.34 ± 0.2 ** | 30.5 ± 0.44 | 24.82 ± 0.09 ** |
| *α*-Phellandrene | 27.28 ± 0.31 | 36.91 ± 0.64 ** | 43.9 ± 1.28 | 30.79 ± 2.32 ** |
| trans-*β*-Ocimene | 23.21 ± 0.16 | 27.5 ± 0.38 ** | 31.59 ± 0.61 | 25.22 ± 0.13 ** |
| cis-*β*-Ocimene | 27.17 ± 0.69 | 39.03 ± 1.32 ** | 49.45 ± 1.68 | 33.68 ± 4.68 ** |
| p-Cymene | 15.54 ± 0.07 | 17.04 ± 0.15 ** | 18.05 ± 0.1 | 16.31 ± 0.07 ** |
| Terpinolene | 22.48 ± 0.09 | 25.47 ± 0.28 ** | 28.08 ± 0.25 | 23.88 ± 0.1 ** |
| *α*-Cedrene | 21.55 ± 0.02 | 22.41 ± 0.06 ** | 23.37 ± 0.02 | 22.95 ± 0.35 |
| (*E,Z*)-allo-Ocimene | 22.31 ± 0.09 | 25.08 ± 0.19 ** | 27.8 ± 0.39 | 23.91 ± 0.06 ** |
| 2,6-Dimethyl-1,3,5,7-octatetraene, *E,E*- | 15.07 ± 0.01 | 15.79 ± 0.02 ** | 16.6 ± 0.02 | 15.89 ± 0.01 ** |
| Rose oxide | 25.52 ± 0.72 | 34.91 ± 1.51 ** | 43.66 ± 0.98 | 33.91 ± 1.52 ** |
| trans-Rose oxide | 8.16 ± 0.28 | 10.71 ± 0.37 ** | 12.73 ± 0.3 | 10.41 ± 0.3 ** |
| Nerol oxide | 3.75 ± 0.14 | 7.16 ± 0.51 ** | 8.78 ± 0.41 | 4.45 ± 0.1 ** |
| trans-linalool oxide(Furanoid) | 2.54 ± 0.87 | 2.09 ± 0.1 | 2.68 ± 0.37 | 2.7 ± 0.43 |
| cis-linalool oxide(Furanoid) | 1.24 ± 0.03 | 1.5 ± 0.06 ** | 1.56 ± 0.06 | 1.51 ± 0.07 |
| *α*-Citral | 9.83 ± 3.83 | 15.56 ± 7.24 | 9 ± 0.45 | 5.79 ± 0.17 ** |
| *β*-Citral | 4.3 ± 0.32 | 5.12 ± 0.72 | 4.76 ± 0.06 | 4.19 ± 0.03 ** |

Asterisk within rows indicates statistically different values between control group and experiment group according to independent sample t-test, *, p < 0.05, **, p < 0.01.

Table S5. Concentrations (μg/kg, mean, n = 3) of glycosylated and free form monoterpenoids in tomato fruits overexpressing *VviERF003*

| **Substrate** | **Concentrations (μg/kg)** | | | | | |
| --- | --- | --- | --- | --- | --- | --- |
|  | glycosylated | | | free | | |
|  | WT | OEE3-1 | OEE3-2 | WT | OEE3-1 | OEE3-2 |
| Linalool |  |  |  | 48.89 ± 7.94 | 144.63 ± 12.23 ** | 186.96 ± 15.86 ** |
| Nerol | 1.66 ± 0.6 | 3.01 ± 0.92 | 3.13 ± 0.22 * | 3.71 ± 0.43 | 8.19 ± 0.7 ** | 9.31 ± 0.24 ** |
| Geraniol |  |  |  | 3.75 ± 0.75 | 6.27 ± 0.16 ** | 7.89 ± 1.45 * |
| *α*-Terpineol |  |  |  | 14.32 ± 1.58 | 46.16 ± 4.64 ** | 61.43 ± 6.34 ** |
| Terpinen-4-ol | 0.56 ± 0.03 | 0.9 ± 0.11 ** | 1.38 ± 0.03 ** | 3.54 ± 0.46 | 6.86 ± 1.09 ** | 5.03 ± 0.87 |
| Citronellol | 10.94 ± 1.42 | 13.33 ± 0.19 * | 12.49 ± 0.24 | 13.06 ± 7.56 | 4.45 ± 0.19 | 3.15 ± 0.32 |
| (±)-Menthol | 5.84 ± 4.55 | 10.95 ± 4.31 | 4.52 ± 1.77 | 7.49 ± 1.82 | 9.9 ± 3.21 | 15.63 ± 8.09 |
| *β*-Myrcene | 56.83 ± 4.2 | 160.78 ± 11.57 ** | 238.09 ± 14.76 ** | 65.21 ± 2.74 | 190.69 ± 16.75 ** | 227.88 ± 27.34 ** |
| cis-*β*-Ocimene | 19.34 ± 1.9 | 51.52 ± 4.92 ** | 77.06 ± 6.32 ** | 24.94 ± 0.24 | 76.1 ± 6.42 ** | 84.39 ± 11.15 ** |
| p-Cymene | 17 ± 1.81 | 24.93 ± 2.32 ** | 29.3 ± 3.4 ** | 60.67 ± 4.53 | 82.37 ± 4.17 ** | 72.36 ± 8.74 |
| Terpinolene | 2.62 ± 0.32 | 7.69 ± 0.79 ** | 11.71 ± 1.43 ** | 4.94 ± 0.09 | 23.03 ± 2.16 ** | 26.28 ± 4 ** |
| (*E*,*Z*)-allo-Ocimene | 3.14 ± 0.33 | 8.74 ± 0.79 ** | 13.09 ± 1.16 ** | 3.99 ± 0.04 | 13.8 ± 1.22 ** | 15.42 ± 2.02 ** |
| trans-linalool oxide(Furanoid) | 2.35 ± 0.37 | 5.1 ± 0.36 ** | 7.62 ± 0.5 ** | 4.36 ± 0.7 | 8.22 ± 0.6 ** | 9.86 ± 0.71 ** |
| *β*-Phellandrene | 62.75 ± 4.84 | 159.55 ± 13.77 ** | 236.15 ± 13.43 ** | ND | ND | ND |
| D-Limonene | 19.72 ± 2.26 | 44.61 ± 3.5 ** | 63.57 ± 4.9 ** | ND | ND | ND |
| *α*-Phellandrene | 15.13 ± 1.68 | 36.88 ± 2.72 ** | 52.98 ± 3.16 ** | ND | ND | ND |
| trans-*β*-Ocimene | 9.12 ± 0.87 | 24.76 ± 2.39 ** | 37.79 ± 3.51 ** | ND | ND | ND |
| Rose oxide | 2.48 ± 0.04 | 5.61 ± 0.43 ** | 3.07 ± 0.07 ** | ND | ND | ND |
| Neo-allo-ocimene | 2.73 ± 0.44 | 7.57 ± 1.07 ** | 11.39 ± 1.6 ** | ND | ND | ND |
| 2,6-Dimethyl-1,3,5,7-octatetraene, *E*,*E*- | 0.63 ± 0.14 | 2.03 ± 0.2 ** | 2.68 ± 0.23 ** | ND | ND | ND |
| cis-linalool oxide(Furanoid) | 1.61 ± 0.27 | 3.68 ± 0.38 ** | 6.56 ± 0.75 ** | ND | ND | ND |
| Nerol oxide | 4.46 ± 0.38 | 9.4 ± 0.63 ** | 9.94 ± 0.36 ** | ND | ND | ND |
| Hotrienol | 1.78 ± 0.16 | 6.21 ± 1.03 ** | 8.36 ± 0.45 ** | ND | ND | ND |
| α-Cedrene | 4.94 ± 2.16 | 5.54 ± 1.18 | 5.17 ± 0.56 | ND | ND | ND |
| p-Menth-1-en-9-al | 3.94 ± 0.47 | 11.14 ± 0.9 ** | 11.84 ± 0.52 ** | ND | ND | ND |
| cis-Isogeraniol | 0.15 ± 0.09 | 0.5 ± 0.05 ** | 0.31 ± 0.07 | ND | ND | ND |
| 1-Terpinenol | ND | ND | ND | 2.73 ± 0.13 | 2.05 ± 0.7 | 2.77 ± 0.27 |
| p-Mentha-1,5-dien-8-ol | ND | ND | ND | 2.96 ± 0.34 | 4.29 ± 0.37 ** | 3.27 ± 0.4 |
| *β*-Citral | ND | ND | ND | 22.95 ± 0.56 | 13.59 ± 0.83 ** | 16.53 ± 1.64 ** |
| *α*-Citral | ND | ND | ND | 155.82 ± 2.53 | 75.95 ± 5.35 ** | 99.88 ± 6.49 ** |

Asterisk within rows indicates statistically different values between WT fruit and overexpressin fruit according to independent sample t-test, *, p < 0.05, **, p < 0.01; ND.: not detected; The blank parts of the data are shown in figure 6.
